# Polymeric nanocapsules prevent oxidation of core-loaded molecules: evidence based on the effects of docosahexaenoic acid and neuroprostane on breast cancer cells proliferation

**DOI:** 10.1186/s13046-015-0273-z

**Published:** 2015-12-21

**Authors:** Jérôme Roy, Liliam Teixeira Oliveira, Camille Oger, Jean-Marie Galano, Valerie Bultel-Poncé, Sylvain Richard, Andrea Grabe Guimaraes, José Mário Carneiro Vilela, Margareth Spangler Andrade, Thierry Durand, Pierre Besson, Vanessa Carla Furtado Mosqueira, Jean-Yves Le Guennec

**Affiliations:** Inserm U1046, UMR CNRS 9214, Physiologie et Médecine Expérimentale du Cœur et des, Muscles – PHYMEDEXP, Université de Montpellier, CHU Arnaud de Villeneuve, Bâtiment Crastes de Paulet, 371 avenue du doyen Gaston Giraud, 34295 Montpellier Cedex 5 Montpellier, France; Laboratório de Desenvolvimento Galênico e Nanotecnologia - CiPharma, Escola de Farmácia, Universidade Federal de Ouro Preto, Minas Gerais, Brazil; Institut des Biomolécules Max Mousseron (IBMM), CNRS UMR 5247, Université de Montpellier, ENSCM, Montpellier, France; Centro Tecnológico CETEC SENAI/FIEMG–Belo Horizonte, Minas Gerais, Brazil; Inserm U1069, Nutrition, Croissance et Cancer, Université François-Rabelais de Tours, Tours, France

**Keywords:** Nanocapsules, Docosahexaenoic, Acid, Neuroprostanes, Polyunsaturated fatty acids, Asymmetrical flow-field-flow-fractionation, Breast carcinoma cells

## Abstract

**Background:**

Nanocapsules, as a delivery system, are able to target drugs and other biologically sensitive molecules to specific cells or organs. This system has been intensively investigated as a way to protect bioactives drugs from inactivation upon interaction with the body and to ensure the release to the target. However, the mechanism of improved activity of the nanoencapsulated molecules is far from being understood at the cellular and subcellular levels. Epidemiological studies suggest that dietary polyunsaturated fatty acids (PUFA) can reduce the morbidity and mortality from breast cancer. This influence could be modulated by the oxidative status of the diet and it has been suggested that the anti-proliferative properties of docosahexaenoic acid (DHA) are enhanced by pro-oxidant agents.

**Methods:**

The effect of encapsulation of PUFA on breast cancer cell proliferation in different oxidative medium was evaluated in vitro. We compared the proliferation of the human breast cancer cell line MDA-MB-231 and of the non-cancer human mammary epithelial cell line MCF-10A in different experimental conditions.

**Results:**

DHA possessed anti-proliferative properties that were prevented by alpha-tocopherol (an antioxidant) and enhanced by the pro-oxidant hydrogen peroxide that confirms that DHA has to be oxidized to exert its anti-proliferative properties. We also evaluated the anti-proliferative effects of the 4(*RS*)-4-F_4t_-neuroprostane, a bioactive, non-enzymatic oxygenated metabolite of DHA known to play a major role in the prevention of cardiovascular diseases. DHA-loaded nanocapsules was less potent than non-encapsulated DHA while co-encapsulation of DHA with H_2_O_2_ maintained the inhibition of proliferation. The nanocapsules slightly improves the anti-proliferative effect in the case of 4(*RS*)-4-F_4t_-neuroprostane that is more hydrophilic than DHA.

**Conclusion:**

Overall, our findings suggest that the sensitivity of tumor cell lines to DHA involves oxidized metabolites. They also indicate that neuroprostane is a metabolite participating in the growth reducing effect of DHA, but it is not the sole. These results also suggest that NC seek to enhance the stability against degradation, enhance cellular availability, and control the release of bioactive fatty acids following their lipophilicities.

## Background

Breast cancer represents one of the major problems for public health. Epidemiological studies have highlighted environmental factors as having a major role in the etiology of breast cancer. Genetic factors are obviously involved in carcinogenesis, but diet, as an environmental factor, is likely to have an influence on health [1], and particularly on tumor emergence [2–4]. For 40 years, differences in breast cancer incidence among women over the world and the influence of migrations on this incidence suggested that environmental factors such as dietary fat may play a role in this disease [5, 6]. Some epidemiological and experimental data have revealed that PUFA, especially ω3 PUFA, inhibit the development and the progression of breast cancer [7]. But this observation is still controversial [8–12]. In vivo studies showed that the long chain ω3 PUFA α-linolenic acid (ALA, 18:3n-3), eicosapentaenoic acid (EPA, 20:5n-3) and docosahexaenoic acid (DHA, 22:6n-3) inhibit the progression of breast cancer [13–15].

The inhibitory effect of n-3 PUFA seems to be correlated with the number of skipped dienes [16]. Several studies observed that the inhibition of mammary cancer cells proliferation was more marked by a treatment with DHA, than with EPA or ALA [17–22]. This suggests that lipid peroxidation could be a prerequisite for PUFA to be active [15, 17, 23–27]. Peroxidation of lipids followed by transformation of the peroxides into secondary metabolites may be a crucial step on the path leading to reduction of cell proliferation rate [28].

DHA had already been formulated in different micro and nanometrical delivery systems, such as liposomes [29] nanoliposomes [30], cyclodextrins-polymer complex [31], zein-prolamine-microcapsules [32] and carbohydrate-based-microcapsules [33]. None of these systems have a fluid oily core acting as reservoir, where PUFA could be easily encapsulated and protected by a polymeric biodegradable wall. Furthermore, no investigations of their effects related to the oxidative status on tumor breast cancer cell proliferation were presented to date.

Nanoencapsulation consists of creating small droplets of liquid (core), which is packed inside a polymeric wall, designed to protect the core from deterioration and release it under desired conditions [34]. Also, polymeric nanocapsules (NC) have been shown to reinforce the biological effects of encapsulated drugs [35–37], by increasing drug efficacy while reducing toxicity [38]. This can be due to a better targeting of the compounds to the cell [39] or/and a better protection of the encapsulated molecules against environmental factors such as light or enzymatic attack during their transit through the digestive tract [40].

In the present study we hypothesized that NC could protect the PUFA against oxidation to reach the breast tumor cells in their active form. The effects of encapsulation of DHA and 4F4t-NeuroP in NC were investigated on mammary cancer cell proliferation following different oxidative status in order to study the PUFA stability and also the ability to act on cells proliferation. Poly-ε-caprolactone (PεCL) nanocapsules filled with the PUFA were designed herein in order to provide delivery systems to be used orally. PεCL polymer is more appropriate to resist to the adverse environment of the gastrointestinal tract (GIT) than other biodegradable polyesters [41]. Furthermore, free-PUFA and PUFANC were tested in different oxidative conditions. Poly-ε-caprolactone (PεCL) NC loaded with DHA or 4(RS)-4-F_4t_-neuroprostane (4F_4T_-NeuroP), an oxygenated metabolite derived from DHA, were prepared by polymer deposition followed by solvent displacement [42]. The NC were characterized by dynamic light scattering (DLS), atomic force microscopy (AFM) and asymmetrical flow field flow fractionation (AsF4) coupled to multi-angle laser light scattering detector (MALLS), DLS and UV–vis detectors. The capacity of NC to protect drugs against oxidation was evaluated in vitro. To do so, we took advantage of the need of DHA to be oxidized in order to inhibit tumor cell proliferation. Also, we evaluated if some pharmacological beneficial effects of NC [37–39, 43] are mediated by a protection of the encapsulated drugs against oxidation. We also tested if 4F4t-NeuroP, the most abundant product of non-enzymatic oxidation of DHA [44] also has anti-proliferative properties.

## Results

### Nanocapsules development and characterization

**Fig. 1:**
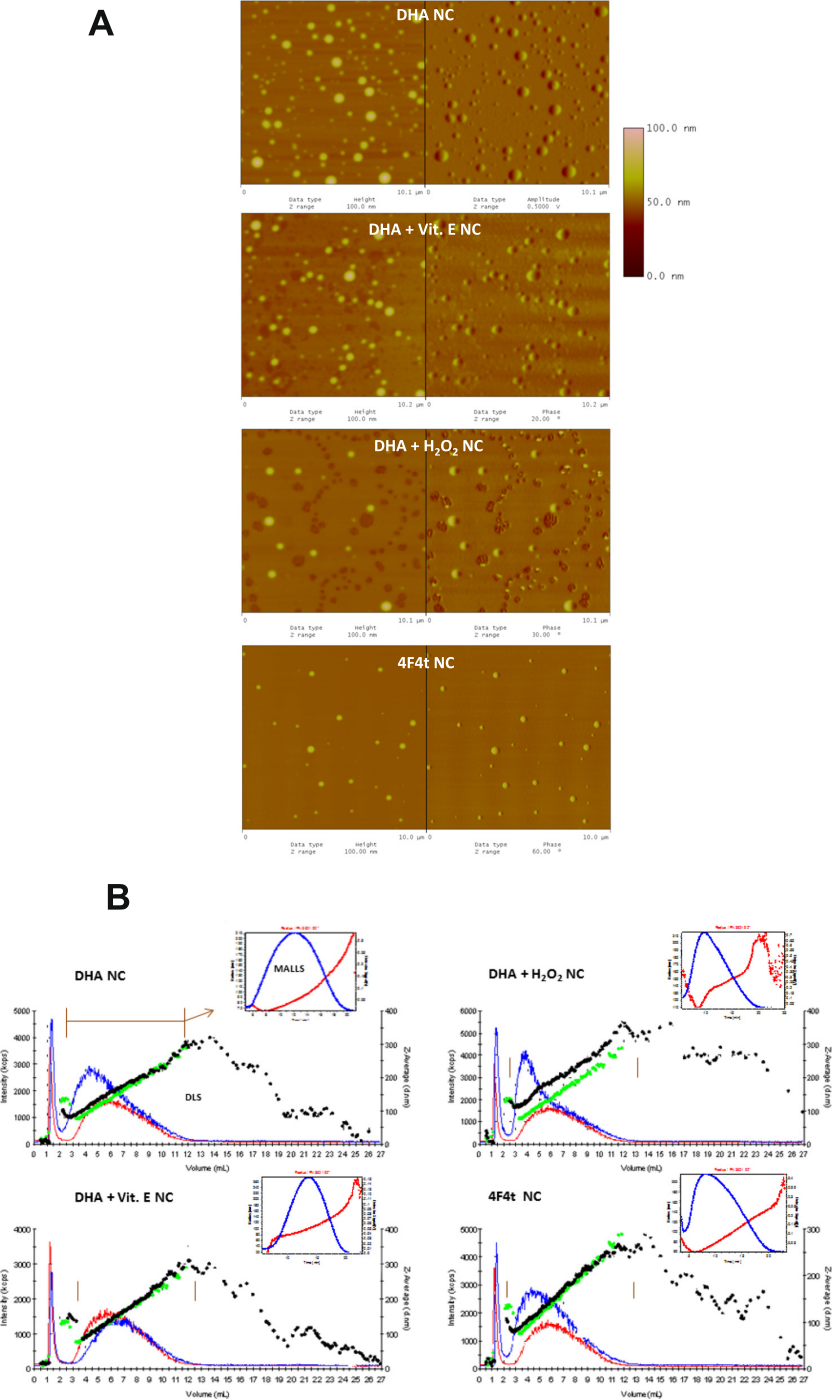
Characterisation of nanocapsules in the different experimental conditions. **a** Atomic Force Microscopy (AFM) images of height (*left*) and phase (*right*) showing the Differences in The PUFA-loaded NC size dispersion and Morphology (scan Sizes are 10×10 μm). **b** Fractograms Of the different PUFA nanocapsules After Asymmetrical-Flow-Field-Flow-Fractionation (AsF4). Black Dots represent Detection by DLS (*Z*-average hydrodynamic diameter) and the blue trace represents UV detection. Green dots (DLS) and red full line (UV-intensities) refer to unloaded PεCL NC for comparison in each graph. The inserts represent the traces of 92o angle analysis from peak selection zones (*brown bars*) where the MALLS Analysis was performed to provide radius of gyration (R*g*, Root mean square radius) (*red Line and left Y axis*) and detector signal intensity in volume (*blue Line and right Y axis*). The First peak at short retention times is the void volume

The DHA and 4F_4t_-NeuroP were encapsulated in the oily core of polymeric (PεCL) NC. Table 1 shows the main physicochemical characteristics of the optimized NC formulations. Using two sizing methods (DLS in liquid state and AFM in argon dried state) for the determination of particle size distribution gave very similar results, with no statistical difference between both sizing methods. This confirmed the colloidal nature of the polymeric NC and the narrow dispersion of the NC population prepared from DHA and 4F_4t_-NeuroP using the polymer deposition followed by solvent displacement method [45]. The accurate mean sizes were measured because NC average diameters were in the best resolution range of DLS and AFM techniques, 3–1000 nm and 0.5–1000 nm, respectively [46]. Mean sizes of 150–180 nm were obtained with the blank-NC, DHA, DHA + VitE and 4F_4t_-NeuroP loaded NC (Table 1) with a monodispersed profile of size distribution. However, a significant increase (*P* < 0.05) in mean size was evidenced by the DLS and AFM for the DHA + H_2_O_2_ NC formulation (Table 1). The general aspect of PUFA core-loaded NC height images in AFM is homogenous and monodisperse, at the exception of DHA + H_2_O_2_ NC (Fig. 1a). The AFM phase images show the leaky and heterogeneous nature of DHA + H_2_O_2_ NC compared with the three other NC formulations. Furthermore, the NC deformation, shrinking and leakage was evidenced under the tip pressure along with the probe scanning in *tapping* mode (not shown). Fractionation of the nanoparticle sample by the AsF4 can be presented in fractograms (Fig. 1b), where the most intense peaks were selected for further analysis of nanoparticles by DLS and MALLS detectors. The fractograms of ASF4 coupled to DLS show that all NC formulations were highly homogenous in size, even with the encapsulation of PUFA compared to unloaded NC filled only with medium chain triglycerides (data not shown). However, AS-F4-MALLS analysis of DHA + H_2_O_2_ NC showed more heterogeneity in size distribution and the presence of larger aggregates (Fig. 1b) compared with DHA NC. All three sizing techniques (AFM, DLS, ASF4-MALLS) evidenced the same increase in sizes after inclusion of hydrogen peroxide in NC formulation compared to DHA NC (Table 1). No significant difference in size was observed after the additional loading of VitE in DHA NC formulation. The summary of the results is shown in Table 1. DLS provides the *Z*-average hydrodynamic diameter and hydrodynamic radius by calculation and the MALLS gives the geometric diameter calculated from the direct determination of gyration radius (mean square root). The ratio R*g*/R*h* allows estimating the shape factor [46]. As a result of fractionation, a significant reduction in mean sizes was observed because larger size and aggregated nanoparticles were removed and only the most abundant NC population were analyzed. The values of the shape factors indicate that blank NC, NC-DHA, NC-DHA + VitE and 4F_4t_-neuroP are soft spheres with values of shape factors closer to 0.977, for a hollow sphere [46]. This is in accordance with a vesicular nature of those NC with oil filled core. On the other hand, DHA + H_2_O_2_ NC differs greatly from the spherical shape, which suggests worm-like structures, with shape factors approximately of 2. This suggests coalescence of DHA + H_2_O_2_ NC and system instability under minimal pressure stress while macroscopic observations of the NC suspension samples in basal condition did not indicate any sign of flocculation or aggregation.

**Table 1:** Physicochemical characterization of the PUFA nanocapsules using different methods before and after sample fractionation

			*Before fractionation*			*After fractionation*	
Formulations	AFM	DLS	Mean	Mean ζ Zeta	AsF4-DLS	AsF4-MALLS	R*g*/R*h*
	mean size	Z-average size	PdI	Potential ± SD (mV)	Z-average	D*g* (nm)	*(shape factor)*
	± SD (nm)^a^	D*h* ± SD (nm)			D*h* (nm)		
NC PCL	193 ± 28	157 ± 3	0.122	−28.9 ± 1.4	124 ± 9	111 ± 1	1.152
NC PCL-DHA	183 ± 26	161 ± 1	0.113	−29.7 ± 0.7	145 ± 31	116 ± 7	1.032
NC PCL-DHA + Vit E	203 ± 42	179 ± 3	0.089	−20.6 ± 0.7^b^	170 ± 25	128 ± 15	0.976
NC PCL-DHA + H2O2	236 ± 59	207 ± 2^b, c^	0.160	−32.4 ± 1.6^c^	144 ± 31	205 ± 13^c^	1.838^c^
NC PCL-4F4t	194 ± 52	161 ± 3	0.111	−29.5 ± 1.6	120 ± 18	112 ± 9	1.218

*ζ* Zeta Potential, *AFM* atomic force microscopy, *AsF4* asymmetrical flow-field-flow- fractionation, *DLS* dynamic light scattering, *PdI* polydispersity index, *Dh* z- verage hydrodynamic diameter, *Dg* mean geometric diameter (D*g* = 2*Rg*0.775), *LDA* Laser Doppler Anemometry, *MALLS* Multi Angle Laser Light Scattering Detector, *PεCL* poly-ε-caprolactone, *Rg* radius of gyration (root mean square radius), *Rh* hydrodynamic radius, *EE* encapsulation efficiency, *PdI* polydispersity index, *Dh* z-average hydrodynamic diameter, *Dg* mean geometric diameter (Dg = Rg*2*0.775), *Rg* radius of gyration (root mean square radius), *Rh* hydrodynamic radius. ^a^determined by the measurements of at least 40 particles in the height images; mean values taken from the selection of the most intense peak in the fractogram. Shape factor calculation (Mathaes et al., 2013). ^b^Significant difference (*p* <0.05) from control formulation; ^c^ Significant difference (*p* <0.05) from non-oxidized

The zeta potentials of NC filled with medium chain triglyceride (blank-NC), Miglyol oil, and of those filled with PUFA were not significantly different, indicating that the PUFA were successfully encapsulated and that they had a weak influence on surface charges (Table 1). In the opposite, when VitE was co-encapsulated with DHA, it induced a significant increase in zeta potential in comparison with DHA encapsulation alone (from − 29.7 ± 0.7 mV to − 20.6 ± 0.7 mV) (Table 1).

### Effects of DHA on cell growth

**Fig. 2:**
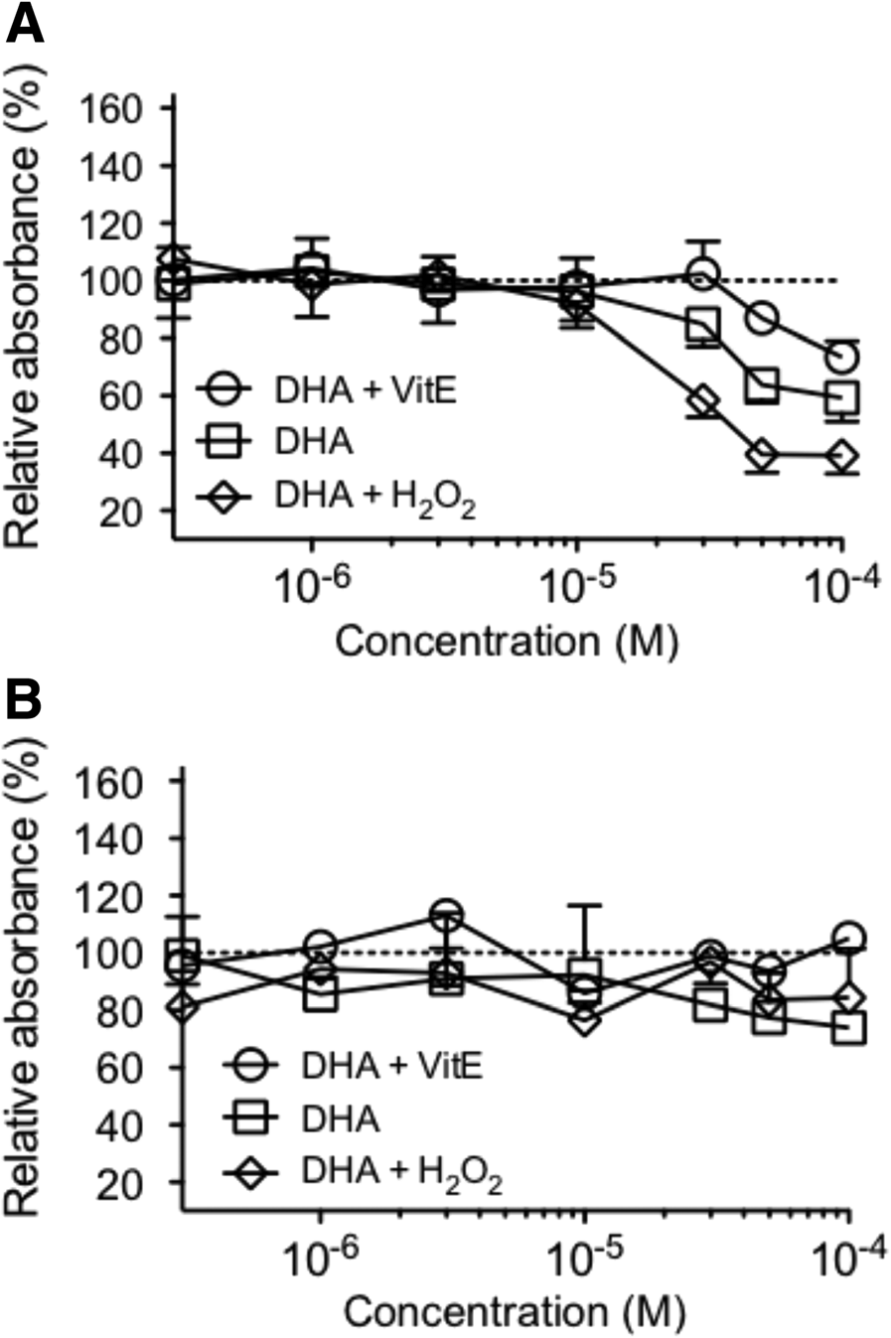
Effect of DHA + VitE, DHA and DHA + H2O2 on the growth of breast carcinoma cell line MDA-MB-231 (**a**) and normal immortalized breast epithelial cells MCF-10A (**b**). The cells were exposed to 0.3, 1, 3, 10, 30, 50, 100 μM and after 4 days, cell proliferation was measured by MTT 0.5 mg/mL method (see Materials and methods). The relative cell proliferation (absorbance of test wells) was expressed as a percentage of the control (100 %) that was not treated with DHA (absorbance of control wells without DHA or with NC alone). Points are expressed as the mean values relative to control for three separate experiments, each set up in triplicate wells

DHA inhibited the growth of MDA-MB-231 cancer cells in a dose-dependent manner (Fig. 2a) while it had no effect on the immortalized epithelial MCF-10A cells (Fig. 2b). The inhibition of cancer cells proliferation by DHA was prevented in the presence of VitE and enhanced with H_2_O_2_ in the medium (Fig. 2a). It has to be noted that the inhibition of cancer cell proliferation does not exceed 55 % at the highest concentration tested (100 μM). The immortalized MCF-10A cells were insensitive to DHA whatever the environmental oxidative status (Fig. 2b).

### Effects of DHA encapsulation on cell proliferation

**Fig. 3:**
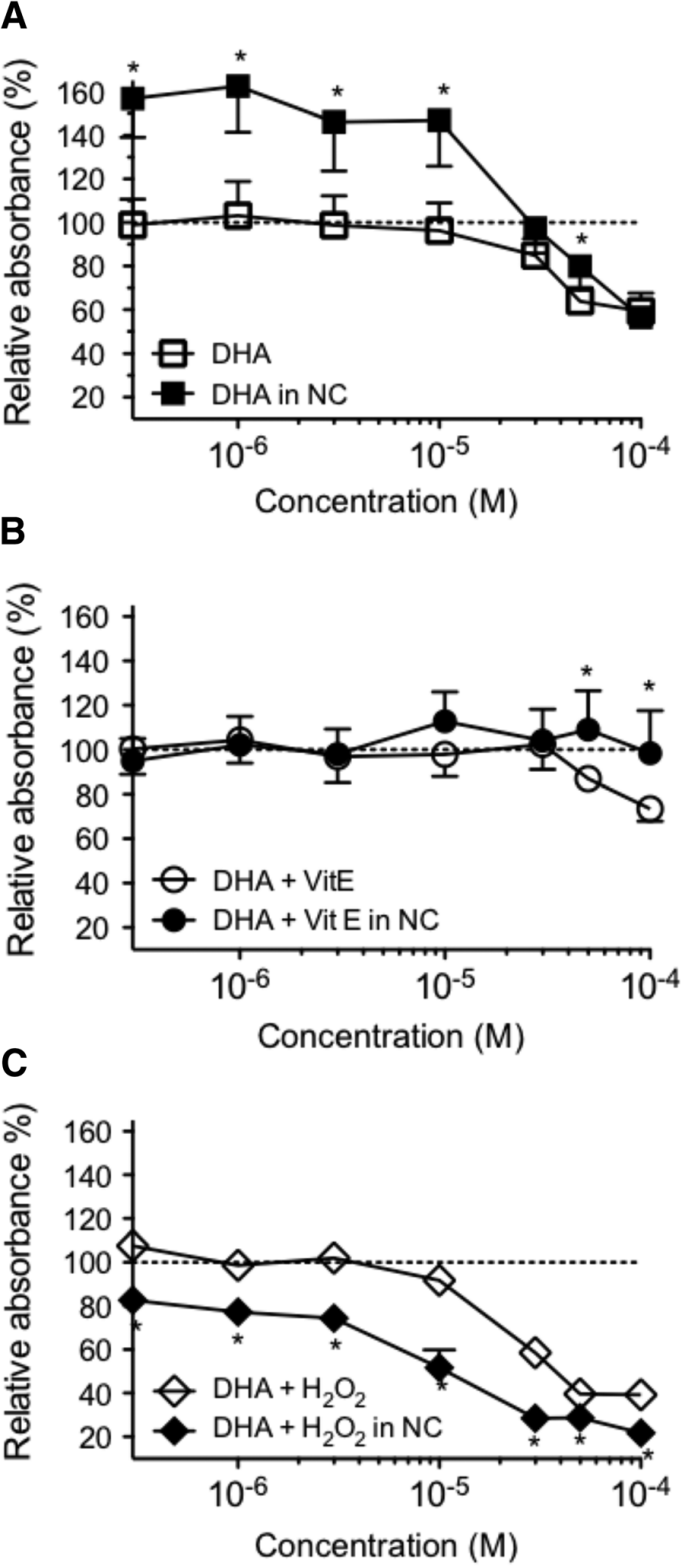
Differential effects of free or encapsulated DHA on the growth the breast carcinoma cell line MDA-MB-231. Cells were exposed to free DHA (**a**), free DHA + VitE (**b**) and free DHA + H2O2 (**c**) over a 0.3–100 μM concentration range or an equivalent volume of ethanol (delivery vehicle) as a control. The same conditions were used with nanocapsules containing the same substances. The MTT assay was performed after 4 days culture. The relative cell proliferation (absorbance of test wells). was expressed as a percentage of the control (line 100 %) that was not treated with DHA (absorbance of control wells without DHA or with NC alone). Results are expressed as absorbance, normalized as percent of control (100 %). * Significantly different between the free and encapsulated substances at the same concentration, at *P* < 0.05. Data points are mean ± SEM of three separate experiments, each one performed in three wells

Encapsulation of DHA alone or in association with an anti-oxidant or a pro-oxidant modified its effects on the proliferation of cancer cell line MDA-MB-231. DHA in NC at concentrations lower than 30 μM increased cell proliferation when compared to free DHA (Fig. 3a). However, above 30 μM both free and encapsulated DHA reduced MDA-MB-231 cell proliferation. In contrast, in the presence of VitE, DHA encapsulation below 50 μM did not modify the proliferation (Fig. 3b) while free DHA at high concentrations (100 μM) reduced cell proliferation by approximately 30 %. With H_2_O_2_ (Fig. 3c) NC potentiated the anti-proliferative properties of DHA at all the concentrations tested.

### Effect of DHA-derived oxygenated metabolites on cell proliferation

**Fig. 4:**
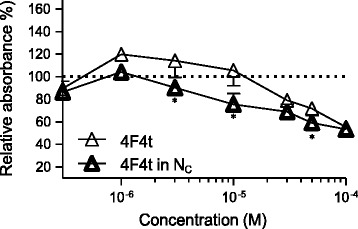
Effect of free or encapsulated 4(*RS*)-4-F4t-neuroprostane on the growth the breast carcinoma cell line MDA-MB-231. Cells were exposed 4 days to 0.3, 1, 3, 10, 30, 50 and 100 μM then cell proliferation was measured by the MTT method (see Materials and methods). The relative cell proliferation (absorbance of test wells) was expressed as a percentage of the control (line 100 %) that was not treated with 4(*RS*)-4-F4t-neuroprostane (absorbance of control wells with vehicle or NC with vehicle). * Significantly different between the free and encapsulated substance at the same concentration, at *P* < 0.05. Data points are mean ± SEM of three separate experiments, each one performed in three wells

The oxidative status (presence of VitE or H_2_O_2_) of the medium surrounding DHA appears to influence the effects of the PUFA on cell proliferation. This suggests that the formation of oxygenated metabolites can be involved. To investigate this hypothesis, we used 4F_4t_-neuroP, an oxygenated metabolite of DHA, which is the most common non-enzymatic oxygenated metabolite of DHA [44]. This metabolite was thus synthesized [47] and tested on cultured cells. Free 4F_4t_-neuroP had a slight potentiating effect on cell proliferation at concentrations up to 10 μM (Fig. 4) in breast cancer cells and had no effect in normal cells (date not shown). Encapsulation of 4F_4t_-neuroP significantly increased (*p* < 0.05) the anti-proliferative effect at concentrations above 1 μM (Fig. 4).

## Discussion

PCL is a suitable biodegradable polymer to resist the GIT acid and alkaline environment compared to more hydrophilic and fast degradable ones, PLA and PLGA [41]. The physicochemical characterization of NC showed that DHA and 4F_4t_-NeuroP were successfully encapsulated in the oily core of this polymeric system. The NC average sizes are in the range of 150–200 nm with no significant difference between DHA and 4F_4t_-NeuroP NC (Table 1). Oppositely, including H_2_O_2_ in the NC formulation induced a strong alteration of the stability of the NC polymeric wall, which consequently induced leakage of the core under minimal pressure stress. The NC filled with VitE showed a reduced zeta potential in modulus, thus a lower charge potential at the NC surface (Table 1). This probably had an influence on the exposure of the negatively charged groups at the NC surface. Oxidation mediated by H_2_O_2_ could lead to the peroxidation of PUFA with the generation of oxygenated metabolites. As oxygenated metabolites generally show high polarity compared to their precursors this would impart negative charges to the NC surface. VitE co-encapsulation with DHA is likely to protect PUFA against peroxidation, thus maintaining lower negative values of zeta potential (Table 1). Also, the polymeric membrane probably increases the protection of the fatty acids against oxidation and also reduces the interaction with cells at selected concentrations.

In this paper, we challenged the hypothesis that NC core-loaded PUFA are protected from peroxidation. Indeed, it is known that the anti-proliferative effects of ω3 PUFA are prevented by VitE [48] while they are enhanced by pro-oxidants [25, 49] that generate oxygenated metabolites. To show that, we took advantage of the well-known effect of those oxygenated metabolites of ω3 PUFA on cancer cells proliferation [20, 25, 26, 48, 50]. In this study we observed an effect of the oxidative status of DHA on a breast cancer cell line in vitro. The oxidative status had a selective role on cancer cell proliferation compared to non-cancer cells (MCF-10A). Similar findings have been reported in the presence of antioxidant, with an increase in the proliferation of cancer cells [15, 51, 52]. Other authors had already reported that DHA impaired breast cancer cell growth and survival by enhancing metabolic stress, particularly by down-regulating total glycolytic metabolism, while not influencing a non-cancer breast epithelial cell line (MCF-10A) [53]. In the first part of the present study, we confirmed a DHA dose-dependent decrease in MDA-MB-231 cells proliferation, a property not found in the non-cancer human mammary epithelial cell line MCF-10A. This effect was reinforced in the presence of H_2_O_2_ and prevented in the presence of VitE. The IC_50_ of free DHA + H_2_O_2_ was approximately 38 μM in cancer cells and not determined in normal cells (no effect at 100 μM), indicating a good selectivity toward cancer cells in vitro.

When encapsulated, DHA at concentrations below 30 μM increased the proliferation of cancer cells, while encapsulation with VitE at comparable concentrations had no effect.

**Fig. 5:**
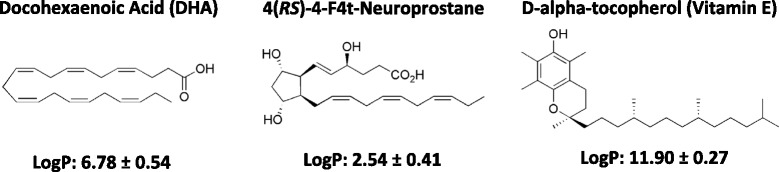
Chemical structures of DHA, 4(RS)-4F4t-neuroprostane and D-alpha-tocopherol (VitE) and the calculated lipophilicity (Log *P*) using ACD/Labs Software v.14.03

DHA is very hydrophobic (log *P* = 6.8) and its release in culture medium is probably delayed and reduced by the high partition coefficient in favor of the NC oily core, even after incubation for 4 days with the cells (Fig. 5). This can explain the reduced DHA anti-proliferative effects on cancer cells. A free fraction of intact DHA (not oxidized) released by the DHA-NC formulation could explain its activity similar to the free air-oxidized DHA (oxidized in the air) at the highest concentrations in vitro (above 50 μM). In the opposite, 4F_4t_-NeuroP is a much more polar fatty acid (log *P* = 2.5) and is probably released from NC faster than DHA, which could explain its improved antiproliferative effect compared to the free 4F_4t_- NeuroP at concentrations of 10–50 μM. The slow release of 4F_4t_-NeuroP from NC into the culture medium probably maintains its activity for longer times, warranting the anti-proliferative effect upon tumor cell. Also, the NC polymeric membrane probably increases the protection of the 4F_4t_-NeuroP against oxidation, which could explain the absence of effect for low concentration for free-4-F_4t_-NeuroP. Thus the difference between the activities of DHA and its non-enzymatic oxygenated metabolite in NC form could be attributed to their difference in lipophilicities (log *P*) and affinities for the NC oily core that further influences their release rate in the cell medium.

The results obtained with encapsulated H_2_O_2_ are difficult to interpret since H_2_O_2_ by itself had an effect on NC. H_2_O_2_ induced degradation of the polymeric NC wall makes difficult the interpretation of the data. The disruption of NC wall probably increases the availability of DHA to be oxidized by H_2_O_2_, which increases the anti-proliferative effect of DHA in the presence of H_2_O_2_. In that case, the investigation of the different mechanism of internalization of nanostructures, similar to *trojan horse* type, via endocytic pathways, could explain an increased delivery of the amount of oxidized DHA metabolites inside cells.

In the opposite to H_2_O_2_, such interactions of DHA with NC were not found in absence and presence of VitE. Knowing that DHA has to be oxidized to inhibit proliferation [25, 49], it is likely that NC protect the fatty acid against oxidation. Indeed, it is known that preparing a DHA solution in a physiological saline solution under the normal atmosphere containing oxygen leads to the oxidation of DHA [54]. Thus, encapsulating DHA in NC did not lead to an enhanced proliferation *per se.* Indeed, it prevented the oxidation of DHA and the associated inhibition of proliferation. The encapsulation of DHA with VitE did not affect cell proliferation when compared to the same experiments without NC since VitE already fully protected free-DHA against oxidation. A better protection of DHA in NC could be observed when huge concentrations of DHA were used (50 and 100 μM) probably because where free-DHA at 50 or 100 μM could not be fully protected by a limiting concentration of VitE.

The mechanism by which secondary products of lipid peroxidation retard or inhibit mammary cancer cell growth processes in vitro and/or in vivo is not certain [53]. Oxygenated metabolites produced by lipid peroxidation are capable of decreasing cell proliferation through damaging cell membranes, by changing membrane lipid composition and structure and/or cytoskeleton assembly [55]. These modifications in the molecular architecture of the membrane can lead to the inactivation of membrane transport systems and/or membrane bound enzymes [56, 57]. Furthermore, secondary products of lipid peroxidation can decrease tumor cell survival by inactivating DNA polymerase reactions [58] forming intramolecular linkages between biomolecules DNA, RNA, and proteins [59], and inhibiting polyamine synthesis. Other studies reported that the induction of apoptosis appears as the major mechanism of action for the products of the peroxidation of PUFA [15, 16, 60, 61]. Recently, it has been shown that the cardiac anti-arrhythmic properties of DHA are due to an oxygenated metabolite of DHA: 4-F_4t_-NeuroP [62]. We tested if this neuroprostane could be responsible for the anti-proliferative effects of DHA and found that, even if it does have some effect, this effect is less pronounced than that of DHA + H_2_O_2_, suggesting that other oxygenated metabolites are involved. Encapsulation of 4-F4t-NeuroP prevented the proliferation-stimulating effects at low doses of the free NeuroP, suggesting that this compound can be further non-enzymatically metabolized.

## Conclusion

In the present paper, we show that NC can prevent the oxidation of encapsulated drugs delaying deteriorative reactions and enhancing chemical stability under different environmental conditions such as cancer. The poly-ε-caprolactone (PεCL) nanocapsules designed for oral route were successfully prepared to encapsulate the PUFA in the oily core. This study shows that they effectively protect PUFAs from oxidative degradation and reduced cellular effects following its lipophilicities. On the other hand in the oxidative environment the NC are destabilized and permit DHA oxidation. Thus the difference between the activities of DHA and its non-enzymatic oxygenated metabolite in NC form could be attributed to their difference in lipophilicities (log *P*) and affinities for the NC oily core that further influences their release rate in the cell medium. The encapsulation process is thus a mean to protect and stabilize the encapsulation of drugs or fatty acids against premature degradation that may occur before it reaches the target after systemic administration.

## Methods

### Reagents and drugs

All reagents for culture cells were obtained from Life Technologies SARL (Cergy Pontoise Cedex, France). Epikuron® 170 (~70 % soy phosphatidylcholine) was a gift from Lucas Meyer (Le Blanc Mesnil, France). Miglyol® 810 N oil (capryc/caprylic triglyceride) was purchased from Hülls (Frankfurt, Germany). The poly-ε-caprolactone (PεCL, average 42,500 Da), Poloxamer®188, DHA, α-tocopherol (vitamin E) and hydrogen peroxide (H_2_O_2_) were provided by Sigma-Aldrich (St. Louis, MO) and the acetone was purchased from Mallinckrodt chemicals (USA). Symplicity® System (Millipore, Bedford, USA) was used to produce Milli-Q water (18.2 MΩ), which was used to prepare nanocapsules and all solutions for this study. The synthesis of 4*(RS)*-4F_4t_-neuroprostane was performed according to our published procedure [47].

### Fatty acid solutions

DHA and 4*(RS)*-4F_4t_-neuroprostane stock solutions (Fig. 5) were prepared in ethanol at a concentration of 100 mM and stored under a nitrogen atmosphere at − 20 °C. The vitamin E stock solution was prepared in chloroform at 1 mM. The hydrogen peroxide stock solution was freshly prepared in MilliQ water at 10 mM.

### Nanocapsules preparation

Poly-ε-caprolactone (PεCL) nanocapsules (NC) were prepared by preformed polymer deposition method followed by solvent displacement as firstly described by [42]. An organic solution was prepared with 60 mg PεCL polymer dissolved in an acetone solution (10 mL) containing 75 mg Epikuron®170, 250 μL Miglyol®810 N and the different concentrations of PUFAs (see Fig. 4). This organic solution was poured into the external aqueous phase (20 mL) containing 75 mg Poloxamer®188 and the mixture was kept under magnetic stirring for 10 min. Solvents were removed under reduced pressure in a rotary evaporator R-215 coupled to vacuum controller (Büchi, Labortechnik AG, Switzerland) to render 10 mL of aqueous colloidal NC suspension of each formulation.

## Characterization of PUFA-loaded nanocapsules

### Size distribution

The *Z*-average hydrodynamic diameter of NC and population polydispersity were determined by dynamic light scattering (DLS) at 25 °C in Beckmann Coulter (Fullerton, USA). Nanosizer N5Plus Analyser at 90° angle detector signals, and in a Malvern Instruments (Worcestershire, UK) Zetasizer Nano PN3702 with non-invasive back-scattering analysis at 173°, coupled *in-line* with the AsF4 equipment. Samples were analyzed after 1:1000 dilution in Milli-Q water. Measurements were performed in triplicate. Values reported are the means ± standard deviation of at least three different batches of each NC formulation.

### Zeta potential

The zeta potential (ζ) was determined by Laser Doppler Anemometry coupled to microelectrophoresis in a Zetasizer Nano PN3702 equipment (Malvern Instruments, Worcestershire, UK). The samples were analyzed after dilution (1:1000) in 1 mM NaCl. Both measurements were performed in triplicate. Values reported are the means ± standard deviation of at least three different batches of each NC formulation.

### Atomic force microscopy

NC samples were also analyzed by scanning-probe microscopy in atomic force mode (AFM). Analyses were performed at atmospheric pressure, at room temperature, on Dimension 3000 atomic force microscope monitored by a Nanoscope IIIa controller (Digital Instruments, Santa Barbara, CA, USA). A droplet (5 μL) of each NC sample was deposited on mica, spread and dried with a stream of argon. The images were obtained in tapping mode, using commercial silicon probes, with a 228 μm-long cantilever, 75–98 kHz resonance frequencies, spring constants of 3.0–7.1 N/m and a nominal tip curvature radius of 5 nm. The scan rate was 1 Hz. Dimensional analyses were performed using the “section analyses” software of the equipment. In order to observe the influence of the probe on NC integrity, regions containing several NC were initially imaged with a large scan size (20 μm). In order to look for any oil leakage, zooming-in at smaller scale (1–2 μm) was then performed on some selected NC, which were then scanned three times. Images were then zoomed-out and these NC were compared, in size and occurrence of oil leakage, with their large-scale NC neighbors. NC were also imaged 24 h after the deposition on mica in order to get information on their integrity with time before fractionation.

### Asymmetrical flow-field-flow-fractionation analysis of nanocapsules

The asymmetrical flow-field-flow-fractionation system (AsF4) used was an AF2000 MT system (Postnova Analytics, Landsberg, Germany), with Solvent degasser PN7520 autosampler (PN5300 model, Postnova Analytics) and Isocratic LC–Pump PN1130 (Postnova Analytics, Landsberg). The channel had a 0.0350 cm thick spacer and was 27.5 cm long (tip to tip) and 2 cm wide. A regenerated cellulose membrane having a nominal cut-off of 10 kDa was fixed over a porous frit wall. Samples diluted in water (1:200) were injected (10–50 μL) and eluted with particle- free Milli-Q water under a channel tip flow of 4.5–0.5 mL/min, for a duration of 35 min. The focusing step consisted of a flow delivered by the injection port of 4.2 mL/min for 2 min. The cross- flow gradient was 4–0 mL/min (35 min). After 35 min a tip flow of 0.5 mL/min was maintained for 20 min to clean up the channel. The system was coupled with a variable wavelength PN3211 ultraviolet spectrophotometric (UV) detector (Postnova Analytics, Landsberg) using a deuterium lamp set at 254 nm, with a PN3621 Multi Angle Laser Light Scattering Detector/MALLS (Postnova Analytics, Landsberg) and with a DLS detector *in line* in Zetasizer PN3702 (Malvern Instruments, UK). The acquisition and processing of data were performed using AF2000 Focus Software Version 1.1.0.23. The geometric radius (R_*g*_) of the NC was calculated using the data from the 15 angles analysis with MALLS detector by applying the coated sphere model [63, 64]. The calculation of the geometric diameter for spherical particles is given by the formula *Dg =* Rg*0.775*2. DLS provides the Z-average hydrodynamic diameter and hydrodynamic radius by calculation and the MALLS gives the geometric diameter (mean square root) calculated from the direct determination of gyration radius. The ratio R*g*/R*h* allows estimating the shape factor [46].

## Cell culture experiments

### Cell culture

Normal immortalized human breast epithelial cell line (MCF-10A) [65] and human breast adenocarcinoma cell line (MDA-MB-231) [66] were purchased from the American Type Culture Collection (LGC Promochem, Molsheim, France). MCF-10A were cultured routinely in DMEM/Ham’s F-12 supplemented with 5 % horse serum, 10 μg/mL insulin, 20 ng/mL epidermal growth factor, 0.5 μg/mL hydrocortisone and 100 ng/mL cholera toxin. MDA-MB-231 cells were cultured in Dulbecco’s modified Eagle’s medium (DMEM, 4.5 g/L D-glucose, 584 mg/L L- glutamine and 3.7 g/L NaHCO_3_) supplemented with 5 % fetal calf serum. Cells were grown at 37 °C in a humidified atmosphere containing 5 % CO_2_. Medium was renewed every day. MCF10A doubling time was 48 hrs *vs.* 24 hrs for MDA-MB-231 cells. Cells were routinely cultured without antibiotics, and were passaged weekly with trypsin-EDTA when attaining 80–100 % confluence.

### Assessment of cell proliferation

Cell proliferation was determined by the tetrazolium salt assay with MTT reagent, [3-(4,5- dimethylthiazol-2-yl)-2,5 diphenyltetrazolium bromide] as described previously [67, 68]. To minimize interference from serum (fetal bovine serum for routine culture of MDA-MB-231 cells and horse serum for routine culture of MCF10A cells), MCF10A cells were adapted to MDA-MB-231 cells medium for 1 week prior to experiments as previously described . MCF-10A and MDA-MB-231 cells were seeded in 96-well plates at a density of 5000 cells per well (15,000 cells/cm^2^). After seeding, cells were incubated for 4 days in 200 μL medium supplemented with increasing concentrations of DHA or 4F_4t_-NeuroP (0.3; 1; 3; 10; 30; 50; 100 μM as previously used [20, 22] or DHA with anti-oxidant (1 μM VitE) or pro-oxidant (1 μM H_2_O_2_). To observe antioxidant or oxidant effect, solutions containing different concentration of DHA with 1 μM VitE or with 1 μM H_2_O_2_ were separately diluted in the media 20 min before application to cells in 96-well plates. In these conditions, DHA was added after VitE or H_2_O_2_. The same protocol was used before encapsulation. We previously described a strategy based on an easily accessible bicyclic precursor to obtain isoprostane derivatives [69]. We applied this strategy to the synthesis of the more complex 4*(RS)*-4-F_4t_-neuroprostane [47].

Several controls were included, which contained an equivalent volume of the delivery vehicle (ethanol, chloroform, VitE-free, H_2_O_2_-free, or blank-NC).

After incubation for 4 days in the different conditions, cells were washed out with PBS to remove the different conditions media and further incubated for 60 min at 37 °C in 200 μL culture medium containing 0.5 mg/mL MTT. Metabolically active cells reduced MTT to purple formazan crystals, which were then solubilized in 200 μL dimethylsulfoxide (DMSO). The absorbance of each well was read at 570 nm in a Spectramax 190 spectrophotometer (Molecular Devices Corporation, Sunnyvale, CA).

For all experiments, products were prepared freshly from stock solution and diluted with growth culture medium. VitE, H_2_O_2_, ethanol and blank NC were evaluated against the cells and no effects on cellular proliferation were observed at the tested doses compared to cell proliferation in the original culture medium.

The relative cell proliferation (absorbance of test wells) was expressed as a percentage of the control (line 100 %) that was not treated with DHA (absorbance of control wells without DHA or blank-NC). Mean values and standard deviation were obtained from the analysis of 5 wells, and the experiments were repeated three times to calculate the standard errors of the experiments.

### Statistical analysis

All data are given as mean ± SEM. Statistical analyses, Kruskal-Wallis non parametric test followed by Dunn’s analysis were performed using GraphPad Prism® (Prism 5 for Mac OS X). A p value of 0.05 or less was considered as statistically significant.

## Abbreviation

ζ: Zeta potential
4F_4T_-NeuroP: 4(RS)-4-F_4t_-neuroprostane
AFM: Atomic force miscroscopy
ALA: α-linolenic acid
AsF4: Asymmetrical flow-field-flow-fractionation system
DHA: Docosahexaenoic acid
EPA: Eicosapentaenoic acid
DLS: Dynamic light scattering
H_2_O_2_: Hydrogen peroxide
MALLS: Multi-angle laser light- scattering detector
MTT reagent: 3-(4,5- Dimethylthiazol-2-yl)-2,5 diphenyltetrazolium bromide
NC: Nanocapsule
PεCL: Poly-ε-caprolactone
PUFA: Dietary polyunsaturated fatty acids
Vitamin E: α-tocopherol

## References

[1] Holmes MD, Willett WC (2004). Does diet affect breast cancer risk?. Breast Cancer Res BCR.

[2] Blanckaert VD, Schelling ME, Elstad CA, Meadows GG (1993). Differential growth factor production, secretion, and response by high and low metastatic variants of B16BL6 melanoma. Cancer Res.

[3] Greenwald P, Clifford CK, Milner JA (2001). Diet and cancer prevention. Eur J Cancer Oxf Engl.

[4] Molokhia EA, Perkins A (2008). Preventing cancer. Prim Care.

[5] Buell P (1973). Changing incidence of breast cancer in Japanese-American women. J Natl Cancer Inst.

[6] Armstrong B, Doll R (1975). Environmental factors and cancer incidence and mortality in different countries, with special reference to dietary practices. Int J Cancer J Int Cancer.

[7] Rose DP, Connolly JM (1999). Omega-3 fatty acids as cancer chemopreventive agents. Pharmacol Ther.

[8] Norrish AE, Skeaff CM, Arribas GL, Sharpe SJ, Jackson RT (1999). Prostate cancer risk and consumption of fish oils: a dietary biomarker-based case–control study. Br J Cancer.

[9] Franceschi S, Favero A, La Vecchia C, Negri E, Dal Maso L, Salvini S (1995). Influence of food groups and food diversity on breast cancer risk in Italy. Int J Cancer J Int Cancer.

[10] Braga C, La Vecchia C, Negri E, Franceschi S, Parpinel M (1997). Intake of selected foods and nutrients and breast cancer risk: an age- and menopause-specific analysis. Nutr Cancer.

[11] Favero A, Parpinel M, Montella M (1999). Energy sources and risk of cancer of the breast and colon-rectum in Italy. Adv Exp Med Biol.

[12] Hannafon BN, Carpenter KJ, Berry WL, Janknecht R, Dooley WC, Ding W-Q (2015). Exosome-mediated microRNA signaling from breast cancer cells is altered by the anti-angiogenesis agent docosahexaenoic acid (DHA). Mol Cancer.

[13] Rose DP, Connolly JM, Rayburn J, Coleman M (1995). Influence of diets containing eicosapentaenoic or docosahexaenoic acid on growth and metastasis of breast cancer cells in nude mice. J Natl Cancer Inst.

[14] Grammatikos SI, Subbaiah PV, Victor TA, Miller WM (1994). Diverse effects of essential (n-6 and n-3) fatty acids on cultured cells. Cytotechnology.

[15] Chajès V, Sattler W, Stranzl A, Kostner GM (1995). Influence of n-3 fatty acids on the growth of human breast cancer cells in vitro: relationship to peroxides and vitamin-E. Breast Cancer Res Treat.

[16] Hawkins RA, Sangster K, Arends MJ (1998). Apoptotic death of pancreatic cancer cells induced by polyunsaturated fatty acids varies with double bond number and involves an oxidative mechanism. J Pathol.

[17] Bégin ME, Ells G, Horrobin DF (1988). Polyunsaturated fatty acid-induced cytotoxicity against tumor cells and its relationship to lipid peroxidation. J Natl Cancer Inst.

[18] Rose DP, Connolly JM (1990). Effects of fatty acids and inhibitors of eicosanoid synthesis on the growth of a human breast cancer cell line in culture. Cancer Res.

[19] Das UN (1990). Gamma-linolenic acid, arachidonic acid, and eicosapentaenoic acid as potential anticancer drugs. Nutr Burbank Los Angel Cty Calif.

[20] Grammatikos SI, Subbaiah PV, Victor TA, Miller WM (1994). n-3 and n-6 fatty acid processing and growth effects in neoplastic and non-cancerous human mammary epithelial cell lines. Br J Cancer.

[21] Thoennes SR, Tate PL, Price TM, Kilgore MW (2000). Differential transcriptional activation of peroxisome proliferator-activated receptor gamma by omega-3 and omega-6 fatty acids in MCF-7 cells. Mol Cell Endocrinol.

[22] Barascu A, Besson P, Le Floch O, Bougnoux P, Jourdan M-L (2006). CDK1-cyclin B1 mediates the inhibition of proliferation induced by omega-3 fatty acids in MDA-MB-231 breast cancer cells. Int J Biochem Cell Biol.

[23] Burns CP, Wagner BA (1991). Heightened susceptibility of fish oil polyunsaturate-enriched neoplastic cells to ethane generation during lipid peroxidation. J Lipid Res.

[24] Gonzalez MJ, Gray JI, Schemmel RA, Dugan L, Welsch CW (1992). Lipid peroxidation products are elevated in fish oil diets even in the presence of added antioxidants. J Nutr.

[25] Gonzalez MJ, Schemmel RA, Dugan L, Gray JI, Welsch CW (1993). Dietary fish oil inhibits human breast carcinoma growth: a function of increased lipid peroxidation. Lipids.

[26] Germain E, Chajès V, Cognault S, Lhuillery C, Bougnoux P (1998). Enhancement of doxorubicin cytotoxicity by polyunsaturated fatty acids in the human breast tumor cell line MDA-MB-231: relationship to lipid peroxidation. Int J Cancer J Int Cancer.

[27] Nøding R, Schønberg SA, Krokan HE, Bjerve KS (1998). Effects of polyunsaturated fatty acids and their n-6 hydroperoxides on growth of five malignant cell lines and the significance of culture media. Lipids.

[28] Roy J, Le Guennec J-Y, Galano J-M, Thireau J, Bultel-Poncé V, Demion M (2015). Non-enzymatic cyclic oxygenated metabolites of omega-3 polyunsaturated fatty acid: Bioactive drugs?. Biochimie.

[29] Tanaka Y, Goto K, Matsumoto Y, Ueoka R (2008). Remarkably high inhibitory effects of docosahexaenoic acid incorporated into hybrid liposomes on the growth of tumor cells along with apoptosis. Int J Pharm.

[30] Rasti B, Jinap S, Mozafari MR, Yazid AM (2012). Comparative study of the oxidative and physical stability of liposomal and nanoliposomal polyunsaturated fatty acids prepared with conventional and Mozafari methods. Food Chem.

[31] Layre A-M, Volet G, Wintgens V, Amiel C (2009). Associative network based on cyclodextrin polymer: a model system for drug delivery. Biomacromolecules.

[32] Torres-Giner S, Martinez-Abad A, Ocio MJ, Lagaron JM (2010). Stabilization of a nutraceutical omega-3 fatty acid by encapsulation in ultrathin electrosprayed zein prolamine. J Food Sci.

[33] Journal of Food Engineering 115 (2013) 443–451 [http://www.ncbi.nlm.nih.gov.gate2.inist.fr/pubmed/?term=Dianzani%2C+M.+U.++Free+radicals%2C+lipid+peroxidation+and+cancer+London%3A+Academic+Press%3A+1982%3B+129-158.]

[34] Petrizzo A, Conte C, Tagliamonte M, Napolitano M, Bifulco K, Carriero V (2015). Functional characterization of biodegradable nanoparticles as antigen delivery system. J Exp Clin Cancer Res CR.

[35] Attili-Qadri S, Karra N, Nemirovski A, Schwob O, Talmon Y, Nassar T (2013). Oral delivery system prolongs blood circulation of docetaxel nanocapsules via lymphatic absorption. Proc Natl Acad Sci U S A.

[36] Branquinho RT, Mosqueira VCF, de Oliveira-Silva JCV, Simões-Silva MR, Saúde-Guimarães DA, de Lana M (2014). Sesquiterpene lactone in nanostructured parenteral dosage form is efficacious in experimental Chagas disease. Antimicrob Agents Chemother.

[37] Mosqueira VCF, Loiseau PM, Bories C, Legrand P, Devissaguet J-P, Barratt G (2004). Efficacy and pharmacokinetics of intravenous nanocapsule formulations of halofantrine in Plasmodium berghei-infected mice. Antimicrob Agents Chemother.

[38] Leite EA, Grabe-Guimarães A, Guimarães HN, Machado-Coelho GLL, Barratt G, Mosqueira VCF (2007). Cardiotoxicity reduction induced by halofantrine entrapped in nanocapsule devices. Life Sci.

[39] Bourdon O, Mosqueira V, Legrand P, Blais J (2000). A comparative study of the cellular uptake, localization and phototoxicity of meta-tetra(hydroxyphenyl) chlorin encapsulated in surface-modified submicronic oil/water carriers in HT29 tumor cells. J Photochem Photobiol B.

[40] Mora-Huertas CE, Fessi H, Elaissari A (2010). Polymer-based nanocapsules for drug delivery. Int J Pharm.

[41] Pohlmann AR, Fonseca FN, Paese K, Detoni CB, Coradini K, Beck RC (2013). Poly(ϵ-caprolactone) microcapsules and nanocapsules in drug delivery. Expert Opin Drug Deliv.

[42] Fessi H, Puisieux F, Devissaguet JP, Ammoury N, Benita S (1989). Nanocapsule formation by interfacial polymer deposition following solvent displacement. Int J Pharm.

[43] De Paula CS, Tedesco AC, Primo FL, Vilela JMC, Andrade MS, Mosqueira VCF (2013). Chloroaluminium phthalocyanine polymeric nanoparticles as photosensitisers: photophysical and physicochemical characterisation, release and phototoxicity in vitro. Eur J Pharm Sci Off J Eur Fed Pharm Sci.

[44] Morrow JD, Roberts LJ (1997). The isoprostanes: unique bioactive products of lipid peroxidation. Prog Lipid Res.

[45] Mosqueira VC, Legrand P, Gref R, Heurtault B, Appel M, Barratt G (1999). Interactions between a macrophage cell line (J774A1) and surface-modified poly (D, L-lactide) nanocapsules bearing poly(ethylene glycol). J Drug Target.

[46] Mathaes R, Winter G, Engert J, Besheer A (2013). Application of different analytical methods for the characterization of non-spherical micro- and nanoparticles. Int J Pharm.

[47] Oger C, Bultel-Poncé V, Guy A, Balas L, Rossi J-C, Durand T (2010). The handy use of Brown’s P2-Ni catalyst for a skipped diyne deuteration: application to the synthesis of a [D4]-labeled F4t-neuroprostane. Chem Weinh Bergstr Ger.

[48] Bégin ME (1987). Effects of polyunsaturated fatty acids and of their oxidation products on cell survival. Chem Phys Lipids.

[49] Cognault S, Jourdan ML, Germain E, Pitavy R, Morel E, Durand G (2000). Effect of an alpha-linolenic acid-rich diet on rat mammary tumor growth depends on the dietary oxidative status. Nutr Cancer.

[50] Takeda S, Horrobin DF, Manku M, Sim PG, Ells G, Simmons V (1992). Lipid peroxidation in human breast cancer cells in response to gamma-linolenic acid and iron. Anticancer Res.

[51] Lhuillery C, Cognault S, Germain E, Jourdan ML, Bougnoux P (1997). Suppression of the promoter effect of polyunsaturated fatty acids by the absence of dietary vitamin E in experimental mammary carcinoma. Cancer Lett.

[52] Colas S, Mahéo K, Denis F, Goupille C, Hoinard C, Champeroux P (2006). Sensitization by dietary docosahexaenoic acid of rat mammary carcinoma to anthracycline: a role for tumor vascularization. Clin Cancer Res Off J Am Assoc Cancer Res.

[53] Mouradian M, Kikawa KD, Dranka BP, Komas SM, Kalyanaraman B, Pardini RS (2014). Docosahexaenoic acid attenuates breast cancer cell metabolism and the Warburg phenotype by targeting bioenergetic function. Mol Carcinog.

[54] Judé S, Bedut S, Roger S, Pinault M, Champeroux P, White E (2003). Peroxidation of docosahexaenoic acid is responsible for its effects on I TO and I SS in rat ventricular myocytes. Br J Pharmacol.

[55] Spector AA, Yorek MA (1985). Membrane lipid composition and cellular function. J Lipid Res.

[56] Spector AA, Burns CP (1987). Biological and therapeutic potential of membrane lipid modification in tumors. Cancer Res.

[57] Farber JL, Kyle ME, Coleman JB (1990). Mechanisms of cell injury by activated oxygen species. Lab Investig J Tech Methods Pathol.

[58] Roubal WT, Tappel AL (1966). Damage to proteins, enzymes, and amino acids by peroxidizing lipids. Arch Biochem Biophys.

[59] Reiss U, Tappel AL (1973). Fluorescent product formation and changes in structure of DNA reacted with peroxidizing arachidonic acid. Lipids.

[60] Menéndez JA, del Mar Barbacid M, Montero S, Sevilla E, Escrich E, Solanas M (2001). Effects of gamma-linolenic acid and oleic acid on paclitaxel cytotoxicity in human breast cancer cells. Eur J Cancer Oxf Engl.

[61] Shen HM, Yang CF, Ding WX, Liu J, Ong CN (2001). Superoxide radical-initiated apoptotic signalling pathway in selenite-treated HepG(2) cells: mitochondria serve as the main target. Free Radic Biol Med.

[62] Roy J, Oger C, Thireau J, Roussel J, Mercier-Touzet O, Faure D (2015). Non-enzymatic lipid mediators, neuroprostanes, exert the anti-arrhythmic properties of docosahexaenoic acid. Free Radic Biol Med.

[63] Giddings JC (1993). Field-flow fractionation: analysis of macromolecular, colloidal, and particulate materials. Science.

[64] Hupfeld S, Ausbacher D, Brandl M (2009). Asymmetric flow field-flow fractionation of liposomes: optimization of fractionation variables. J Sep Sci.

[65] Li J, Liu J, Li P, Mao X, Li W, Yang J (2014). Loss of LKB1 disrupts breast epithelial cell polarity and promotes breast cancer metastasis and invasion. J Exp Clin Cancer Res CR.

[66] Matuskova M, Kozovska Z, Toro L, Durinikova E, Tyciakova S, Cierna Z (2015). Combined enzyme/prodrug treatment by genetically engineered AT-MSC exerts synergy and inhibits growth of MDA-MB-231 induced lung metastases. J Exp Clin Cancer Res CR.

[67] Petronzi C, Festa M, Peduto A, Castellano M, Marinello J, Massa A (2013). Cyclohexa-2,5-diene-1,4-dione-based antiproliferative agents: design, synthesis, and cytotoxic evaluation. J Exp Clin Cancer Res CR.

[68] Mosmann T (1983). Rapid colorimetric assay for cellular growth and survival: application to proliferation and cytotoxicity assays. J Immunol Methods.

[69] Oger C, Brinkmann Y, Bouazzaoui S, Durand T, Galano J-M (2008). Stereocontrolled access to isoprostanes via a bicyclo[3.3.0]octene framework. Org Lett.

